# Mediterranean-Style Diet and Birth Outcomes in an Urban, Multiethnic, and Low-Income US Population

**DOI:** 10.3390/nu13041188

**Published:** 2021-04-03

**Authors:** Dong Keun Rhee, Yuelong Ji, Xiumei Hong, Colleen Pearson, Xiaobin Wang, Laura E Caulfield

**Affiliations:** 1 Center for Human Nutrition, Department of International Health, The Johns Hopkins Bloomberg School of Public Health, Baltimore, MD 21205, USA; drhee3@alumni.jh.edu; 2 Department of Maternal and Child Health, Peking University School of Public Health, Beijing 100191, China; yuelong.ji@bjmu.edu.cn; 3 Center on the Early Life Origins of Disease, Department of Population, Family and Reproductive Health, The Johns Hopkins Bloomberg School of Public Health, Baltimore, MD 21205, USA; xhong3@jhu.edu (X.H.); xwang82@jhu.edu (X.W.); 4 Department of Pediatrics, Boston University School of Medicine and Boston Medical Center, Boston, MA 02118, USA; colleen.pearson@bmc.org

**Keywords:** nutrition, maternal diet, Mediterranean diet, diet counseling, pregnancy, birth outcomes, preterm birth, low birth weight, African Americans, public health

## Abstract

Findings on the role of Mediterranean-style diet (MSD) on duration of pregnancy and birth weight have been inconsistent and based largely on Non-Hispanic white populations, making it unclear as to whether they could extend to African Americans who are at a higher risk of unfavorable birth outcomes. Our study addresses this gap using a large urban, multiethnic, predominantly low-income cohort of mother-infant dyads from Boston, MA, USA. Dietary information was obtained via food frequency questionnaires; health information including birth outcomes were extracted from medical records. A Mediterranean-style diet score (MSDS) was formulated based on intake history, and linear and log-binomial regressions were performed to assess its association with birth outcomes. After adjustment, the lowest MSDS quintile from the overall sample was found to be associated with an increased relative risk (RR) of overall preterm birth (RR 1.18; 95% CI: 1.06–1.31), spontaneous preterm birth (1.28; 1.11–1.49), late preterm birth (1.21; 1.05–1.39), and low birth weight (1.11; 1.01–1.22), compared to the highest quintile. The findings were similar for the African American sample. Our study adds to the current understanding of the diet’s influence on birth outcomes by demonstrating that adherence to MSD may improve birth outcomes for African American women.

## 1. Introduction

Preterm birth (PTB), or birth before 37 completed weeks of pregnancy, is the leading cause of death among children under the age of five years and affects more than fifteen million infants annually worldwide [1]. Despite the decreasing trend in PTB rate over the past decade in the United States (US), one in ten infants were born prematurely in 2019; related medical expenditure was estimated at $6 billion in 2013 nationwide [2,3]. Low birth weight (LBW), which is defined as newborn weight under 2500 g, is linked to PTB, fetal growth restriction, or both. Some of the adverse health consequences of PTB or LBW for children include increased risk of death or various forms of neurological, respiratory, and developmental disabilities [4,5,6]. Mothers who delivered PTB or LBW babies are also more likely to suffer from anxiety and decline in mother-to-infant attachment, which in turn can negatively impact the child’s development [7,8].

Many socioeconomic and behavioral risk factors for PTB and LBW have been identified. These include extreme young or advanced maternal age, non-optimal weight status prior to pregnancy, low income or educational attainment, primiparity, and tobacco use [9,10,11,12]. Associations between the risk of PTB and LBW and newborn sex have also been substantiated [13,14,15]. The role of diabetes and hypertension, as well as genetic influences on these adverse birth outcomes, cannot be discounted, either [13,16,17,18,19,20]. Importantly, there is strong evidence that African Americans may be especially vulnerable to the risks of PTB and LBW [21,22]. Though the reasons for this elevated risk is incompletely understood today, available data suggest that the individuals’ stress from the experiences of interpersonal and institutional racism, and the inequities of access to and quality of peri-conceptional and antenatal maternal health care, may play a role [23].

There is growing evidence that maternal dietary patterns can affect the risk of pregnancy outcomes. For example, the risk may decrease with a high intake of vegetables, fruits, and whole grains, or low intake of fast food and added sugars during pregnancy [24,25]. This intake pattern is similar to a Mediterranean-style diet (MSD), which, despite lacking a uniform definition in terms of its specific food items, intake frequency and amount, generally encourages a variety of food groups including cereals, vegetables, fruits, legumes, beans, dairy, eggs, and fish, depending on the cultural and socio-economic contexts of countries [26,27,28,29,30]. Nevertheless, many studies have reported that a greater adherence to MSD during pregnancy appears to reduce the risk of PTB and LBW [25,31,32,33,34,35]. Importantly, however, most have been based on predominantly Non-Hispanic white populations from the developed countries, and it is unclear as to whether their conclusions could extend to minority women including African Americans who are at particularly high risk of these adverse birth outcomes [36]. In this study, we used a prospective cohort study to assess the association between birth outcomes and maternal adherence to MSD in a sample of predominantly urban, low-income African American and non-African American women.

## 2. Materials and Methods

### 2.1. Cohort Design

The parent study, the Boston Birth Cohort (BBC), has been described in previous publications [37,38]. Briefly, enrollment has been on-going in the majority-minority inner city of Boston, MA since 1998 and includes mothers who delivered singleton live births at the Boston Medical Center (BMC). The cohort is enriched by a higher proportion of PTB and LBW than the respective national figures of about 10% and 6% for the past decade and half [39,40]. Exclusion criteria for enrollment included multiple-gestation pregnancy, pregnancy by in-vitro fertilization, PTB due to trauma, and newborns with major birth defects or congenital chromosomal abnormalities. Within 24 to 72 h of delivery, mothers provided written informed consent for participation and were interviewed by trained research staff using a standardized questionnaire to record their sociodemographic and other relevant characteristics. Clinical information and birth outcomes were abstracted from medical records. The cohort design and methods were approved by the Institutional Review Boards of BMC and the Johns Hopkins Bloomberg School of Public Health prior to the data collection phase. This observational study was conducted in accordance with the Declaration of Helsinki and is registered on clinicaltrials.gov (NCT03228875).

### 2.2. Dietary Assessment

An assessment of dietary intake during pregnancy was completed during early postpartum via a qualitative food frequency questionnaire (FFQ), which is a closed-ended dietary history collection method used to assess how often the respondent ate over a specific period [41]. A reformatted version of the original FFQ that was used in our study was created for reference (Figure S1). Mothers were asked to report their weekly frequency of consumption of numerous food groups during pregnancy. The food groups ultimately selected for analysis were: green vegetables, orange vegetables, fruits, meat, fish, eggs, beans, rice, dairy, and wheat (pasta, bread and cereal). Questions regarding consumption of shellfish, soy, seeds, peanuts, tree nuts, and juice, were added as an update to FFQ and therefore not considered during analyses. The response options for intake frequency were: none, less than one day, one to two days, three to five days, six to seven days, and “I don’t know”. 99.4% of the participants responded to at least one of the items in the FFQ, and 87.6% completed all items.

**Figure 1:**
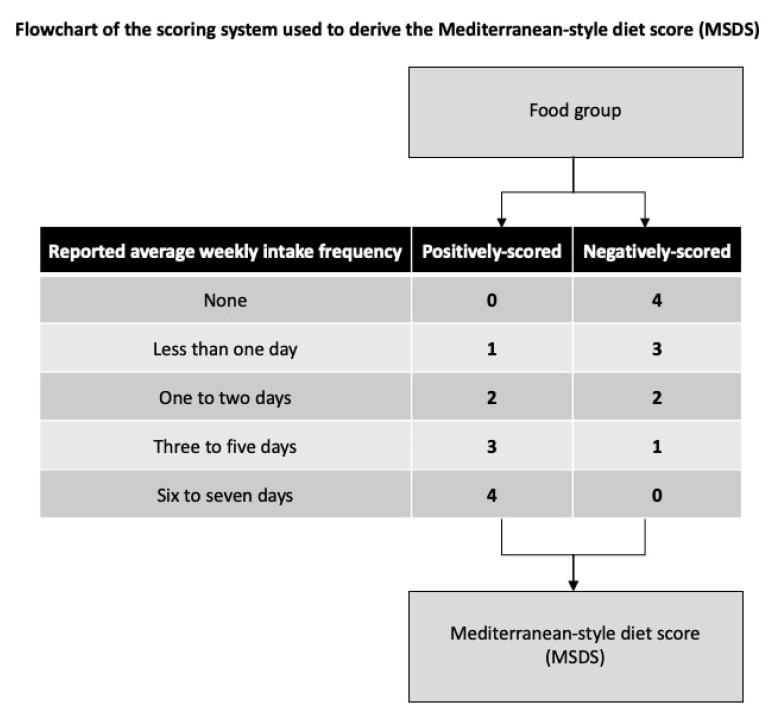
A flow chart of the scoring system used to derive the Mediterranean-style diet score. Each of the food groups was either positively or negatively scored based on the mother’s report of her average weekly intake frequencies during pregnancy. All foods were positively scored except meat, which was negatively scored.

The primary exposure was MSDS assigned to each mother, based on their FFQ responses. Calculating the MSDS requires food groups to be either positively or negatively scored based on the mother’s report of her average weekly intake frequencies during pregnancy. All foods were positively scored except meat, which was negatively scored. For positively-scored foods, we gave a score of 0 to “none”, 1 to “less than one day”, 2 to “one to two days”, 3 to “three to five days”, and 4 to “six to seven days”, and did the reverse for meat (Figure 1). Missing and “I don’t know” responses were imputed with the modal frequency of consumption of that food group for the respondent’s age and racial group. This method of imputation was used to help minimize the risk of bias in the data [42]. Individual scores were then summed to create each participant’s MSDS, with higher scores indicating greater adherence to MSD. For analyses, we identified and compared quintiles of the MSDS with the fifth quintile (i.e., high MSD adherence) as reference.

### 2.3. Assessment of Covariates

Twelve covariates were selected because of their known association with our birth outcomes of interest based on the past literature as explained earlier. These included the maternal age in years, pre-pregnancy body mass index or BMI (body weight in kilograms divided by height in meters squared), parity (number of prior live births), race or ethnicity (African American, Non-Hispanic white, Hispanic, Asian, Haitian, Cape Verdian, Pacific Islander, mixed-race, or other), education level (no school/elementary school, some secondary school, high school graduate or equivalent, some college, or college degree and above), and smoking status during pregnancy (smoking anytime from six months before pregnancy to delivery, restricted to the use of cigarettes, cigars, pipe tobacco, chewing tobacco, or snuff). Overall, 90.9% of the respondents fully completed the demographic questionnaire. Clinical variables included the child’s sex and presence or absence of each major maternal complications: diabetes (diabetes mellitus and gestational diabetes mellitus), chronic hypertension prior to pregnancy, mild or severe preeclampsia, eclampsia, and HELLP syndrome. The research staff successfully abstracted all of the clinical items for 98.0% of the respondents. 

We imputed the missing data for these covariates (the proportion of which for each covariate did not exceed 6.8% across the entire study sample) with the modal frequency of response to each respective covariate for the respondent’s age and racial groups (except for the continuous variables such as maternal age, parity and pre-pregnancy BMI, for which the mean imputation was used). Other potentially relevant variables such as the annual household income and gestational hypertension were not considered due to their proportion of missing responses at greater than 10%.

### 2.4. Assessment of Outcomes

The gestational age in weeks and birth weight in grams from medical records were used to determine each newborn’s specific birth outcomes. PTB was defined as less than 37 completed weeks of gestation, further categorized as spontaneous or medically-induced, and as late PTB (34–36 weeks) or early PTB (less than 34 weeks). LBW was defined as birth weight under 2500 g. The criteria for intrauterine fetal growth—AGA (appropriate for gestational age; weight in the 10–89th percentile), SGA (small for gestational age; weight less than the 10th percentile), and LGA (large for gestational age; weight greater than or equal to the 90th percentile)—were also identified in accordance with the previously published protocols [43,44]. The full extent of data on birth outcomes was abstracted for all participants except one whose baby’s birth weight record was missing. We imputed this missing datum with the mean value for the respondent’s age and racial groups, and assigned a value for the LBW status as applicable.

### 2.5. Statistical Analysis

Univariate analyses using x^2^ test were performed to compare the baseline characteristics of mothers by the individual MSDS quintiles. The primary analyses were based on the sample with imputed data. We assessed the robustness of data by running two parallel analyses with samples comprised of (1) “original data” and (2) “complete-case data” (in which any participant with “I don’t know” or missing response was dropped). Maternal age was re-categorized as: “less than 21”, “between 21 and 30”, and “greater than 30” years of age, and primiparity was identified (y/n). Women were classified based on their pre-pregnancy BMI as: “less than 18.5” (underweight), “between 18.5 and 24.9” (normal), “between 25 and 29.9” (overweight), and “greater than or equal to 30” kg/m^2^ (obese). For analyses, “African American” also included “Haitian”, and those identified as “Asian”, “Cape Verdian”, “Pacific Islander”, “Mixed-race”, and “Other” were grouped as “Other”. Finally, maternal education was grouped as (1) “no school/elementary school” or “some secondary school”, (2) high school or equivalent, and (3) “some college” or “college degree and above”.

For the primary analyses, we assessed the associations between individual MSDS quintiles and birth outcomes—gestational age, overall and categories of PTB, birth weight, LBW, and categories of fetal growth—with the fifth quintile treated as reference. For the subgroups of PTB and fetal growth, term birth and AGA were treated as reference, respectively, to assess the risk of unfavorable birth outcomes relative to their optimal counterparts while allowing for the comparison of the risks between the MSD adherence quintiles. Linear regression was used for continuous outcomes, and log-binomial regression was used for the categorical outcomes. The covariates were adjusted for in the analyses unless the initial univariate analyses revealed that they were not confounders; gestational age was also adjusted for when assessing the birth weight outcomes. For the purpose of adjustment, we collapsed pre-pregnancy BMI to consider overweight/obesity (y/n) and created a binary variable based on the presence of any blood pressure-related complication (chronic hypertension, preeclampsia, eclampsia or HELLP syndrome). The analyses were performed for the overall, African American, and non-African American samples.

Using the overall sample, the unadjusted association between raw MSDS and overall PTB, LBW risks was visualized using kernel density plots. Associations between raw MSDS and spontaneous PTB and LBW risks were visualized using a locally weighted scatterplot smoothing method, stratified by African American and non-African American samples. Finally, adjusting for other covariates as applicable, stratified analyses were performed to assess a covariate’s potential as an effect modifier on four birth outcomes whose associations with MSDS were statistically significant (overall PTB, spontaneous PTB, late preterm births, and LBW). Forest plots were produced for two of these as visual aids. All analyses were performed using Stata Version 14.2 (StataCorp LP, College Station, TX, USA). Statistical significance was set at *p* < 0.05 and all tests were 2-tailed.

## 3. Results

The baseline characteristics of mothers (*n* = 8507) were compared between the MSDS quintiles (Table 1). Mothers in the first quintile (*n* = 2173) were more likely to be younger, non-Hispanic white, and to have less education overall compared to those in any other quintile. They were also more likely to report entering pregnancy with BMI of 30 kg/m^2^ or more, to be nulliparous, have diabetes, and to have smoked during pregnancy.

The baseline characteristics were also compared with those from the original data and the complete-case data (Tables S1 and S2). A flowchart was created to graphically represent the changes in sample size at each step of the data-cleaning process (Figure S2). There were no substantial differences in the sample distribution characteristics between the three datasets except for chronic hypertension. The difference was additionally apparent on parity and diabetes statuses between the imputed and complete-case datasets.

Shown in Table 2 are associations between MSDS quintiles and birth outcomes overall and by racial groups, after covariate adjustments. For all three samples, being in the first quintile (i.e., the lowest MSD adherence), relative to the fifth quintile 5 (i.e., the highest MSD adherence), was consistently associated with a significantly greater risk of overall PTB. The risk of spontaneous PTB, late PTB, and LBW was also significantly elevated for the overall and African American samples but not for the non-African American sample, though the direction of the association was consistent.

**Figure 2:**
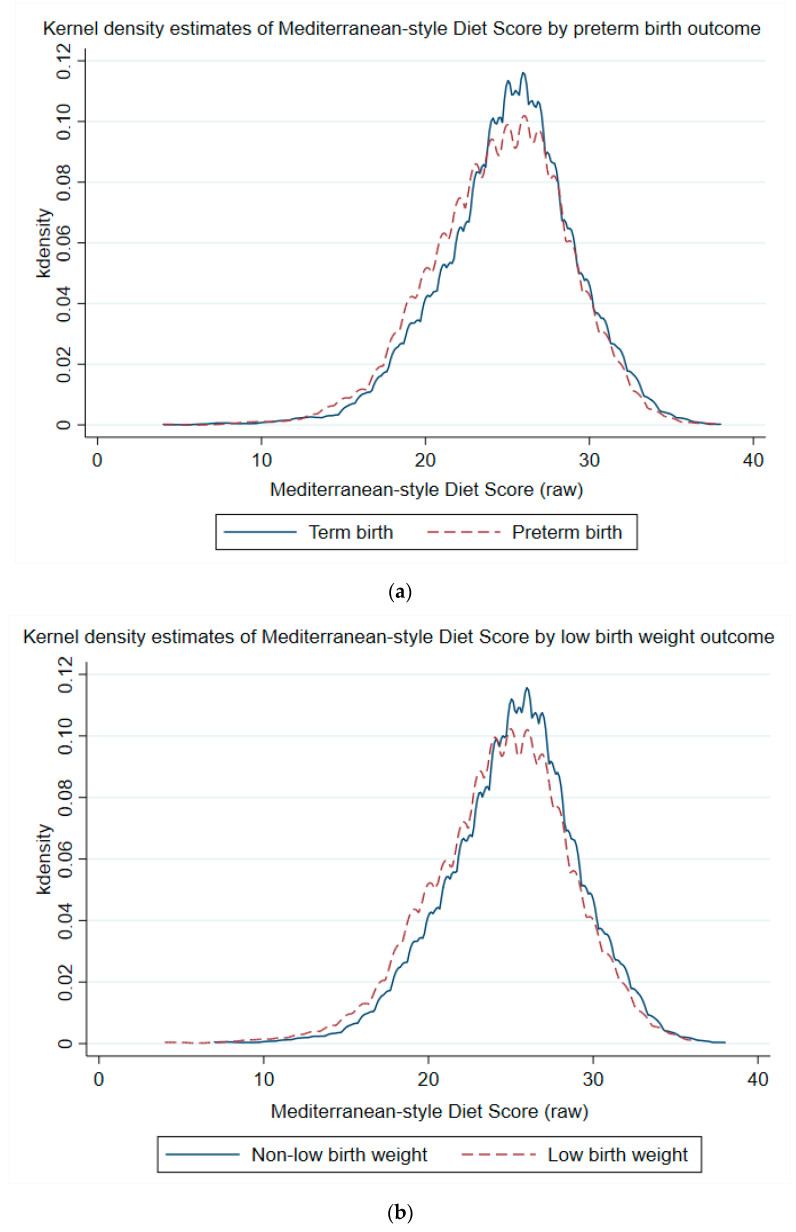
(**a**) Kernel density plots of Mediterranean-style diet score by birth outcomes. Shown are outcomes related to duration of pregnancy, with blue curve for term birth and red dashed curve for overall preterm birth. No adjustments were made for these plots. The *x*-axis represents the raw aggregate Mediterranean-style diet score. (**b**) Kernel density plots of Mediterranean-style diet score by birth outcomes. Shown are outcomes related to birth weight, with blue curve for non-low birth weight and red dashed curve for low birth weight. No adjustments were made for these plots. The *x*-axis represents the raw aggregate Mediterranean-style diet score.

**Figure 3:**
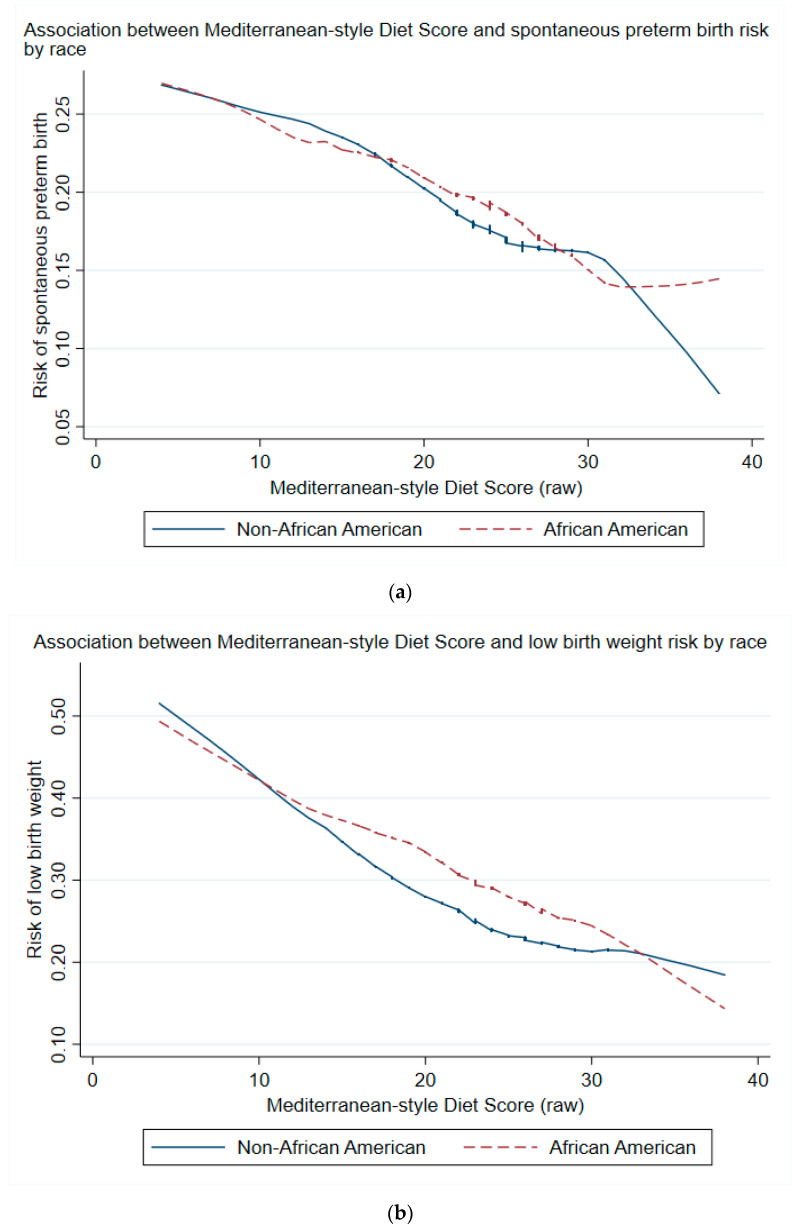
(**a**) Locally weighted scatterplot smoothing of spontaneous preterm birth outcome by MSDS (raw) stratified by race (blue curve for non-African American, red dashed curve for African American). Shown are smoothed curves provided by a weighted quadratic least squares regression over the respective relative risk values. No adjustments were made for this plot. *X*-axis represents the raw aggregate Mediterranean-style diet score. (**b**) Locally weighted scatterplot smoothing of low birth weight outcome by MSDS (raw) stratified by race (blue curve for non-African American, red dashed curve for African American). Shown are smoothed curves provided by a weighted quadratic least squares regression over the respective relative risk values. No adjustments were made for this plot. *X*-axis represents the raw aggregate Mediterranean-style diet score.

When treated as a continuous variable, maternal MSDS was lower for those delivering preterm or LBW as opposed to term or non-low birth weight, respectively (Figure 2a,b). MSDS was negatively associated with risk of PTB and LBW for both African American and non-African American groups (Figure 3a,b). The trends in reduction of risk were similar between groups, notwithstanding the statistical noise toward the lower ends of the curves.

**Figure 4:**
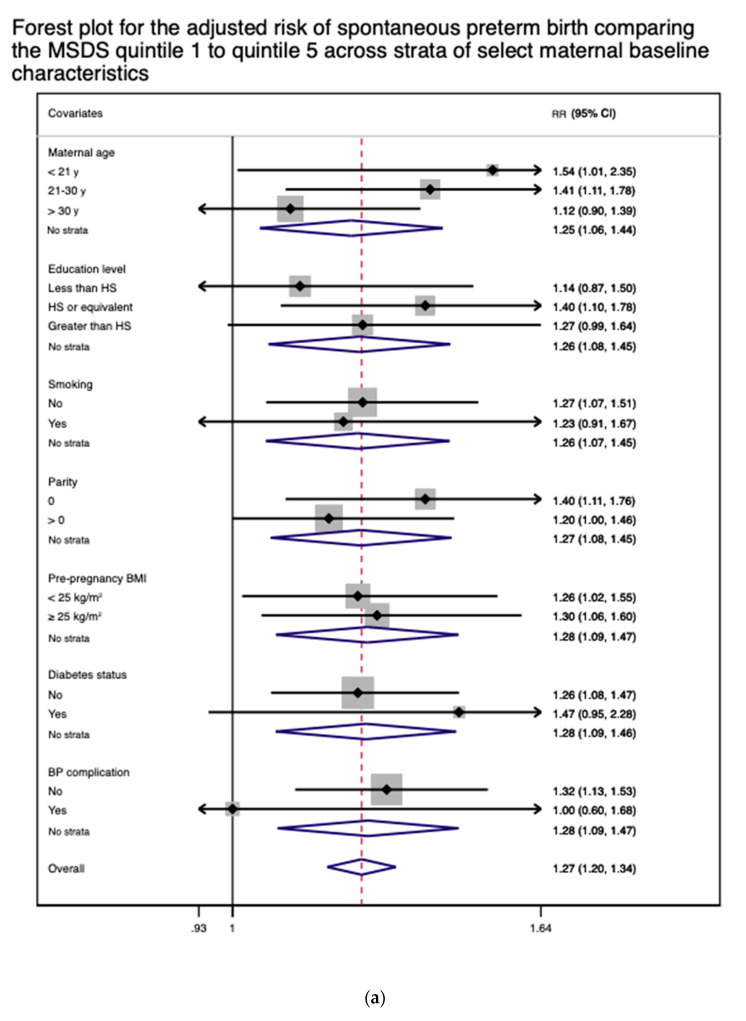

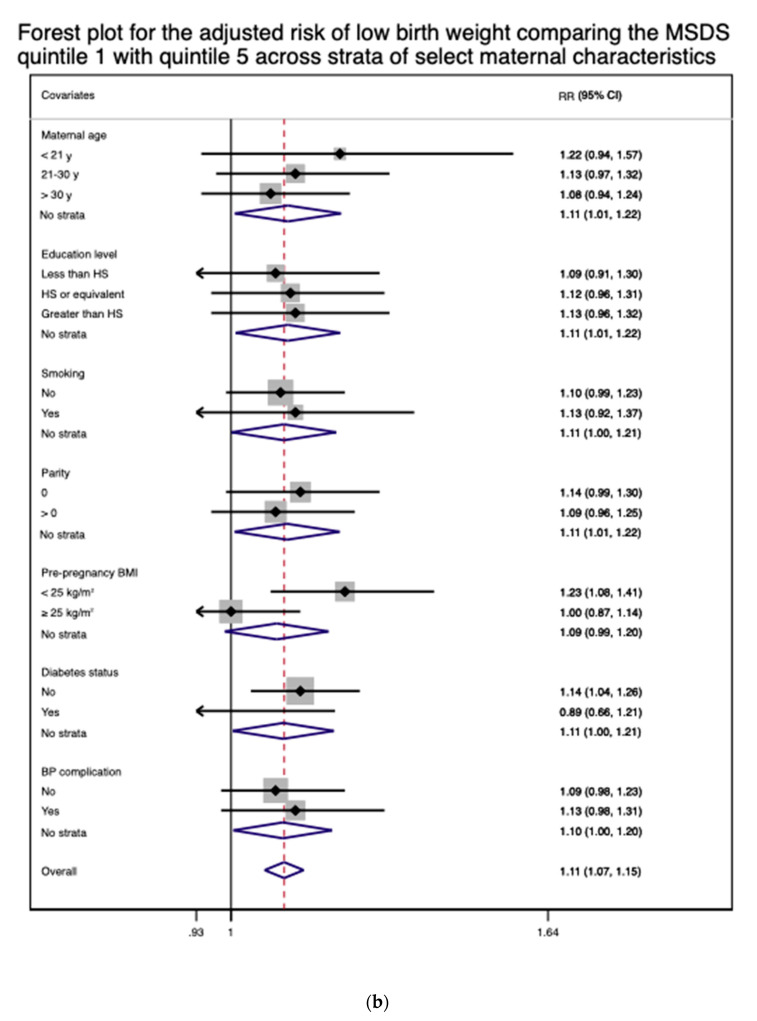
(**a**) A forest plot for the adjusted relative risk (RR) of spontaneous preterm birth comparing the first MSDS quintile with the fifth quintile across the strata of select maternal characteristics. The results were adjusted for all applicable covariates except for child sex. (**b**) A forest plot for the adjusted relative risk (RR) of low birth weight comparing the first MSDS quintile with the fifth quintile across the strata of select maternal characteristics. The results were adjusted for all applicable covariates except for child sex.

The results of stratified analyses suggested that there is no effect modification on overall PTB, spontaneous PTB, late PTB, and LBW by any of the baseline covariates (Table S3), with the 95%-confidence intervals of all outcomes by covariates overlapping with the 95%-confidence intervals of the respective outcomes from the primary analysis (Table 2). This observation was visually apparent in the forest plots for spontaneous PTB and LBW (Figure 4a,b).

## 4. Discussion

In this large urban, multiethnic, predominantly low-income cohort, low adherence to MSD was significantly associated with greater risk for unfavorable birth outcomes. After adjusting for eight maternal sociodemographic and clinical variables, mothers in the first quintile of the overall sample were at 18% greater risk of overall PTB (and 28% for spontaneous PTB, 21% for late PTB relative to term birth) and 11% greater risk of LBW when compared to those in the fifth quintile. For the African American sample, the adjusted excess risk for overall PTB and was at 20% (and 39% for spontaneous PTB, 25% for late PTB relative to term birth); it was 14% for LBW. These figures were not significantly different from those of the non-African American sample, which were 17% for overall PTB (and 20% for spontaneous PTB, 18% for late PTB relative to term birth); the excess risk of LBW for the non-African American group was 5%. For all three sample groups, associations between MSD adherence and fetal growth were statistically nonsignificant, though the direction of the associations was generally consistent and away from growth restriction. These results are generally consistent with prior research indicating that greater adherence to MSD is associated with reduced risk of adverse birth outcomes, particularly preterm delivery [25,31,32,33,34,35].

These clear associations from our study are unsurprising given that the maternal nutritional status and diet are long recognized for their influence on duration of pregnancy and size at birth [36,45,46]. Across published studies, however, findings on the associations between specific dietary patterns during pregnancy and birth outcomes have been inconsistent, including those involving MSD. Some non-US cohort studies have made contradictory observations: the odds of PTB were negatively associated with greater maternal adherence to MSD in one study but not in the other [31,47]. In terms of LBW, Hajianfar et al. found that adhering to a “Western diet” including processed meat and sweets was associated with elevated risk, whereas no association between MSD and LBW was apparent according to Edmond et al. [48,49]. Finally, Parlapani et al. demonstrated that low adherence to MSD was associated with greater risk of intrauterine growth restriction, whereas Saunders et al. suggested there was no such relationship [33,50].

Heterogeneity in the results of associations of MSD and birth outcomes in the literature may be due to a number of factors including: (1) sample characteristics, (2) covariates controlled for in each respective article’s statistical modeling, and (3) dietary assessment techniques. The BBC is a cohort of mothers with mostly low-income background and a diverse racial and ethnic profile. As such, BBC is unique amongst the aforementioned studies which are based predominantly on healthy Non-Hispanic white populations. Our analyses based on the African American demographic group comprising a large proportion of the cohort may have produced more profound effect sizes, given that this population is typically at an elevated risk of the outcomes of interest to our study. This, in turn, highlights the importance of furthering the research into the underlying factors that may be related to the greater vulnerability of African American women, though the above studies are also from outside the US and make a direct comparison difficult [23]. 

In addition, differences between the aforementioned studies in choosing which variables to adjust for in their models are likely contributing to the lack of research consensus on this subject. For instance, pre-pregnancy BMI was adjusted for in many studies while the use of prenatal vitamin supplements was considered infrequently. Of note, this type of inconsistency in adjustment strategies was also recognized by a separate systematic review that recently assessed the literature on the association between MSD and birth outcomes [36]. Lastly, researchers using different diet scoring systems can lead to great variability in the associations with outcomes of interest. This is partly due to the lack of strong consensus on the definition of MSD in the literature, which affords researchers a degree of freedom to devise their own scoring system [26]. As consequence, adherence of the diet scores can range greatly and result in poor correlation between the indices and heterogeneity in the literature [51]. In our study, available dietary data did not completely match those of the studies whose scoring systems were already validated, including those of Panagiotakos et al. [27,52]. Given this, we decided to base our diet scoring system on our understanding of the MSD characteristics, which was ultimately similar to one used by Panagiotakos et al. [29,52].

Our study has several strengths. We found consistent and significant associations between MSD adherence and various birth outcomes in the overall sample and across racial groups. Given the high proportion of African American sample in BBC, our findings might be extendable to low-income African Americans in other US settings. Our study is also bolstered by relatively large sample size and by the high quality of data obtained by trained research staff using standardized protocols. In addition, to mitigate unmeasured confounding, we adjusted for many known sociodemographic and clinical variables that are pertinent to our study. Lastly, we improved the credibility of our single imputation method for the missing values and “I don’t know” responses by using each item’s mean or modal frequency of items that were matched for respondent’s age and racial groups.

Our study has some limitations as well. Firstly, this is an observational study and study participants were asked about their antenatal intake pattern after delivery. This raises the question about whether the mothers correctly recalled their general intake pattern spanning the previous ten months, especially given that surveys are subject to social desirability bias. Secondly, the FFQ gathered data on intake frequency but not quantity. Thirdly, because of limitations in the diet questionnaire, we were unable to use a validated method for scoring, and we did not adjust our food groups or scores for energy intake. In addition, some of the other potentially relevant information, including the diet prior to pregnancy and physical activity history, was not available. Finally, as BBC, by design, had a relatively high proportion of PTB compared to the general US population, caution is needed to generalize our findings to other study populations with different characteristics.

## 5. Conclusions

In conclusion, in our multi-ethnic and predominantly low-income sample, low adherence to MSD was significantly associated with elevated risk of various categories of PTB, LBW, and the subgroups. The excess risk was also significant and similar in magnitude for the African American sample. Our findings add to the literature that maternal dietary patterns influence birth outcomes and suggest the need for further research on how to improve maternal diet during pregnancy, especially, in high-risk vulnerable populations and in low-resource communities.

## Figures and Tables

**Table 1:** Baseline characteristics of mothers in the Boston Birth Cohort, overall sample stratified by MSDS quintiles.

	*N* (%)					
Baseline Characteristics	Total	Quintile 1	Quintile 2	Quintile 3	Quintile 4	Quintile 5
**No.**	8507	2173 (25.5)	1481 (17.4)	1832 (21.5)	1536 (18.1)	1485 (17.5)
**Maternal age (years) ^#^**						
<21	1270 (14.9)	440 (20.3)	227 (15.3)	293 (16.0)	168 (10.9)	142 (9.6)
21–30	3940 (46.3)	1042 (48.0)	703 (47.5)	845 (46.1)	710 (46.2)	640 (43.1)
>30	3297 (38.8)	691 (31.8)	551 (37.2)	694 (37.9)	658 (42.8)	703 (47.3)
**Race or ethnicity ^#^**						
African American *	4030 (47.4)	1021 (47.0)	719 (48.6)	848 (46.3)	750 (48.8)	692 (46.6)
Non-Hispanic White	1005 (11.8)	395 (18.2)	176 (11.9)	194 (10.6)	135 (8.8)	105 (7.1)
Hispanic	2423 (28.5)	535 (24.6)	423 (28.6)	575 (31.4)	460 (30.0)	430 (29.0)
Other ^†^	1049 (12.3)	222 (10.2)	163 (11.0)	215 (11.7)	191 (12.4)	258 (17.4)
**Education level ^#^**						
Less than HS	2688 (31.6)	774 (35.6)	456 (30.8)	612 (33.4)	457 (29.8)	389 (26.2)
HS or equivalent	2879 (33.8)	744 (34.2)	513 (34.6)	595 (32.5)	520 (33.9)	507 (34.1)
Greater than HS	2940 (34.6)	655 (30.1)	512 (34.6)	625 (34.1)	559 (36.4)	589 (39.7)
**Child’s sex**						
Female	4272 (50.2)	1114 (51.3)	750 (50.6)	898 (49.0)	761 (49.5)	749 (50.4)
Male	4235 (49.8)	1059 (48.7)	731 (49.4)	934 (51.0)	775 (50.5)	736 (49.6)
**Ever smoked during pregnancy ^#^**						
No	6869 (80.8)	1509 (69.4)	1215 (82.0)	1505 (82.2)	1325 (86.3)	1315 (88.6)
Yes	1638 (19.3)	664 (30.6)	266 (18.0)	327 (17.9)	211 (13.7)	170 (11.5)
**Parity ^||^**						
0	3661 (43.0)	986 (45.4)	621 (41.9)	806 (44.0)	624 (40.6)	624 (42.0)
>0	4846 (57.0)	1187 (54.6)	860 (58.1)	1026 (56.0)	912 (59.4)	861 (58.0)
**Pre-pregnancy BMI (kg/m^2^) ^#^**						
<18.5	370 (4.4)	111 (5.1)	74 (5.0)	68 (3.7)	69 (4.5)	48 (3.2)
18.5–24.9	3902 (45.9)	963 (44.3)	661 (44.6)	870 (47.5)	699 (45.5)	709 (47.7)
25–29.9	2629 (30.9)	625 (28.8)	462 (31.2)	555 (30.3)	503 (32.8)	484 (32.6)
≥30	1606 (18.9)	474 (21.8)	284 (19.2)	339 (18.5)	265 (17.3)	244 (16.4)
**Diabetes ^||^**						
None	7624 (89.6)	1903 (87.6)	1338 (90.3)	1650 (90.1)	1398 (91.0)	1335 (89.9)
GDM	576 (6.8)	169 (7.8)	93 (6.3)	112 (6.1)	99 (6.5)	103 (6.9)
DM	307 (3.6)	101 (4.7)	50 (3.4)	70 (3.8)	39 (2.5)	47 (3.2)
**Chronic hypertension prior to pregnancy ^||^**						
No	8065 (94.8)	2055 (94.6)	1386 (93.6)	1757 (95.9)	1453 (94.6)	1414 (95.2)
Yes	442 (5.2)	118 (5.4)	95 (6.4)	75 (4.1)	83 (5.4)	71 (4.8)
**Preeclampsia ^||^**						
None	7675 (90.2)	1939 (89.2)	1334 (90.1)	1676 (91.5)	1384 (90.1)	1342 (90.4)
Mild	317 (3.7)	74 (3.4)	59 (4.0)	71 (3.9)	68 (4.4)	45 (3.0)
Severe	515 (6.1)	160 (7.4)	88 (5.9)	85 (4.6)	84 (5.5)	98 (6.6)
**Eclampsia**						
No	8489 (99.8)	2168 (99.8)	1478 (99.8)	1828 (99.8)	1535 (99.9)	1480 (99.7)
Yes	18 (0.2)	5 (0.2)	3 (0.2)	4 (0.2)	1 (0.1)	5 (0.3)
**HELLP syndrome**						
No	8442 (99.2)	2155 (99.2)	1477 (99.7)	1819 (99.3)	1521 (99.0)	1470 (99.0)
Yes	65 (0.8)	18 (0.8)	4 (0.3)	13 (0.7)	15 (1.0)	15 (1.0)

Abbreviations: HS, high school; BMI, body mass index; GDM, gestational diabetes mellitus; DM, diabetes mellitus. *p*-values derived using χ^2^ test. Percentages may not add to 100% due to rounding. * Included African Americans and Haitians. ^†^ Included Asian, Cape Verdian, Pacific Islander, mixed-race, and others. *p*-values (χ^2^ test): ^||^ (≤0.05); ^#^ (<0.001).

**Table 2:** Adjusted association between MSDS quintiles and pregnancy outcomes in the Boston Birth Cohort, relative to quintile 5.

Outcome		Effect Size (95% CI)			
	*N* (%)	Quintile 1	Quintile 2	Quintile 3	Quintile 4
**Overall sample**	8507	N/A	N/A	N/A	N/A
**Gestational age**					
Continuous (weeks) *	N/A	−0.34 (−0.55–−0.13) ^#^	−0.13 (−0.35–0.10)	−0.01 (−0.22–0.20)	−0.07 (−0.29–0.15)
<37 weeks ^†^	2316 (27.2)	1.18 (1.06–1.31) ^¶^	1.05 (0.94–1.18)	0.96 (0.86–1.08)	1.05 (0.93–1.18)
Spontaneous	1528 (18.0)	1.28 (1.11–1.49) ^¶^	1.12 (0.95–1.31)	1.05 (0.90–1.23)	1.14 (0.97–1.33)
Medically-induced	788 (9.3)	1.01 (0.85–1.21)	0.95 (0.77–1.15)	0.82 (0.67–1.00)	0.90 (0.74–1.10)
Preterm subgroups ^†^					
≥37 weeks	6191 (72.8)	1.00 [Reference]	1.00 [Reference]	1.00 [Reference]	1.00 [Reference]
34–36 weeks	1504 (17.7)	1.21 (1.05–1.39) ^¶^	1.05 (0.90–1.23)	0.93 (0.80–1.09)	1.06 (0.91–1.24)
<34 weeks	812 (9.5)	1.20 (0.99–1.46)	1.03 (0.83–1.28)	0.98 (0.80–1.21)	1.01 (0.81–1.25)
**Birth weight**					
Continuous (g) *	N/A	−43.54 (−74.51–−12.57) ^¶^	−41.07 (−74.16–−7.98) ^||^	−13.71 (−45.15–17.72)	−9.95 (−42.60–22.69)
Low birth weight ^†^	2221 (26.1)	1.11 (1.01–1.22) ^||^	1.16 (1.05–1.28) ^¶^	1.07 (0.97–1.18)	1.03 (0.93–1.14)
**Fetal growth ^†^**					
10–89th %iles	6649 (78.2)	1.00 [Reference]	1.00 [Reference]	1.00 [Reference]	1.00 [Reference]
<10th %ile	777 (9.1)	1.16 (0.96–1.39)	1.20 (0.99–1.46)	1.06 (0.88–1.29)	1.11 (0.91–1.35)
≥90th %ile	1080 (12.7)	0.86 (0.70–1.05)	0.97 (0.70–1.05)	0.87 (0.71–1.07)	0.89 (0.72–1.10)
**African American sample ^†^**	4030	N/A	N/A	N/A	N/A
**Gestational age**					
Continuous (weeks) *	N/A	−0.31 (−0.63–0.01)	−0.18 (−0.53–0.16)	0.07 (−0.26–0.41)	0.06 (−0.28–0.40)
<37 weeks ^†^	1160 (28.8)	1.20 (1.03–1.39) ^||^	1.13 (0.96–1.33)	0.98 (0.83–1.15)	1.04 (0.89–1.23)
Spontaneous	742 (18.4)	1.39 (1.12–1.71) ^¶^	1.26 (1.00–1.58)	1.18 (0.94–1.49)	1.16 (0.92–1.47)
Medically-induced	418 (10.4)	0.94 (0.74–1.18)	0.95 (0.73–1.22)	0.69 (0.52–0.91) ^¶^	0.86 (0.67–1.12)
Preterm subgroups ^†^					
≥37 weeks	2870 (71.2)	1.00 [Reference]	1.00 [Reference]	1.00 [Reference]	1.00 [Reference]
34–36 weeks	692 (17.2)	1.25 (1.01–1.54) ^||^	1.18 (0.94–1.48)	0.98 (0.77–1.23)	1.10 (0.88–1.39)
<34 weeks	468 (11.6)	1.18 (0.92–1.51)	1.06 (0.81–1.39)	0.93 (0.71–1.22)	0.92 (0.69–1.21)
**Birth weight**					
Continuous (g) *	N/A	−59.66 (−103.83–−15.50) ^¶^	−49.57 (−96.68–−2.46) ^||^	−0.04 (−45.43–45.34)	−20.15 (−66.70–26.40)
Low birth weight ^†^	1147 (28.5)	1.14 (1.01–1.29) ^||^	1.17 (1.03–1.33) ^||^	1.01 (0.88–1.16)	1.11 (0.97–1.27)
**Fetal growth ^†^**					
10–89th %iles	3161 (78.4)	1.00 [Reference]	1.00 [Reference]	1.00 [Reference]	1.00 [Reference]
<10th %ile	360 (8.9)	1.22 (0.93–1.58)	1.18 (0.89–1.56)	0.96 (0.72–1.28)	1.19 (0.89–1.57)
≥90th %ile	509 (12.6)	0.84 (0.63–1.13)	0.89 (0.65–1.23)	0.91 (0.68–1.23)	0.93 (0.69–1.26)
**Non-African American sample**	4477 (52.6)	N/A	N/A	N/A	N/A
**Gestational age**					
Continuous (weeks) *	N/A	−0.35 (−0.62–−0.09) ^¶^	−0.04 (−0.33–0.25)	−0.07 (−0.34–0.20)	−0.18 (−0.46–0.11)
<37 weeks ^†^	1156 (25.8)	1.17 (1.01–1.36) ^||^	0.97 (0.82–1.15)	0.95 (0.80–1.11)	1.05 (0.89–1.24)
Spontaneous	786 (17.6)	1.20 (0.98–1.47)	0.99 (0.79–1.24)	0.95 (0.76–1.18)	1.11 (0.89–1.38)
Medically-induced	370 (8.3)	1.12 (0.85–1.47)	0.92 (0.67–1.25)	0.95 (0.71–1.27)	0.92 (0.68–1.24)
Preterm subgroups ^†^					
≥37 weeks	3321 (74.2)	1.00 [Reference]	1.00 [Reference]	1.00 [Reference]	1.00 [Reference]
34–36 weeks	812 (18.1)	1.18 (0.98–1.43)	0.95 (0.77–1.18)	0.90 (0.74–1.11)	1.03 (0.84–1.27)
<34 weeks	344 (7.7)	1.22 (0.89–1.68)	0.98 (0.68–1.40)	1.04 (0.75–1.45)	1.15 (0.81–1.62)
**Birth weight**					
Continuous (g) *	N/A	−17.06 (−60.25–26.13)	−23.65 (−70.08–22.79)	−17.13 (−60.73–26.48)	6.11 (−39.70–51.93)
Low birth weight ^†^	1074 (24.0)	1.05 (0.91–1.21)	1.12 (0.96–1.31)	1.10 (0.95–1.27)	0.93 (0.79–1.09)
**Fetal growth ^†^**					
10–89th %iles	3489 (77.9)	1.00 [Reference]	1.00 [Reference]	1.00 [Reference]	1.00 [Reference]
<10th %ile	417 (9.3)	1.07 (0.84–1.38)	1.19 (0.92–1.55)	1.13 (0.88–1.46)	1.03 (0.78–1.36)
≥90th %ile	571 (12.8)	0.91 (0.69–1.19)	1.06 (0.80–1.41)	0.85 (0.64–1.13)	0.87 (0.64–1.17)

Covariates adjusted for are: maternal age, race, educational level, smoking status, parity, pre-pregnancy BMI, diabetes status, and blood pressure complications during pregnancy. Continuous birth weight outcome was derived additionally adjusting for the gestational age, and low birth weight outcome was derived additionally adjusting for the overall preterm birth. For the subgroups of PTB and fetal growth, term birth and AGA were treated as reference, respectively, to assess the risk of unfavorable birth outcomes relative to their optimal counterparts while allowing for the comparison of the risks between the MSD adherence quintiles. * Linear regression. ^†^ Log-binomial regression (relative risk). *p*-values: ^||^ (≤0.05); ^¶^ (<0.01); ^#^ (<0.001).

## Data Availability

The data presented in this study are available on request from the corresponding author. The data are not publicly available due to restrictions of informed consent and the requirement of IRB review and approval.

## Supplementary Materials

The following are available online at https://www.mdpi.com/article/10.3390/nu13041188/s1, Figure S1: Reformatted food frequency questionnaire used for Boston Birth Cohort; Figure S2: Flowchart of 8507 mother-infant dyads enrolled in the Boston Birth Cohort; Table S1: Baseline characteristics of mothers in the Boston Birth Cohort, overall sample and stratified by MSDS quintiles (original data); Table S2: Baseline characteristics of mothers in the Boston Birth Cohort, overall sample and stratified by MSDS quintiles (complete-case data), Table S3: Adjusted stratified analyses on the association between MSDS quintiles and birth outcomes in the Boston Birth Cohort, relative to quintile 5 (overall sample).
