# Supplementary material for: Mediterranean-Style Diet and Birth Outcomes in an Urban, Multiethnic, and Low-Income US Population
Source: Nutrients. 2021 Apr 3;13(4):1188. doi: 10.3390/nu13041188 (PMC8066173; doi:10.3390/nu13041188)
Supplement: Supplementary file 1 [file nutrients-13-01188-s001.pdf]

nutrients

MDPI

Article

Mediterranean-Style Diet and Birth Outcomes in an Urban, Multiethnic, and Low-Income US Population

Dong Keun Rhee [1], Yuelong Ji [2], Xiumei Hong [3], Colleen Pearson [4], Xiaobin Wang [5] and Laura E Caulfield [6,*]

Reformatted food frequency questionnaire used for Boston Birth Cohort						
Question: During this pregnancy, on average, how often did you eat or drink following foods per week?						
	Weekly intake frequency					
Food group	None	<1 day	1-2 days	3-5 days	6-7 days	Don't know
Green vegetables	O	O	O	O	O	O
Orange vegetables (carrots, squash, etc.)	O	O	O	O	O	O
Fruits	O	O	O	O	O	O
Meats	O	O	O	O	O	O
Shellfish	O	O	O	O	O	O
Fish	O	O	O	O	O	O
Eggs	O	O	O	O	O	O
Cow's milk / Dairy products / Cheese	O	O	O	O	O	O
Beans	O	O	O	O	O	O
Rice	O	O	O	O	O	O
Wheat (pasta, bread, cereal)	O	O	O	O	O	O
Soy / Tofu	O	O	O	O	O	O
Seeds (sesame, sunflower, pumpkin)	O	O	O	O	O	O
Calcium-fortified juice	O	O	O	O	O	O
Peanuts	O	O	O	O	O	O
Tree nuts	O	O	O	O	O	O

Figure S1. Reformatted food frequency questionnaire used for Boston Birth Cohort.

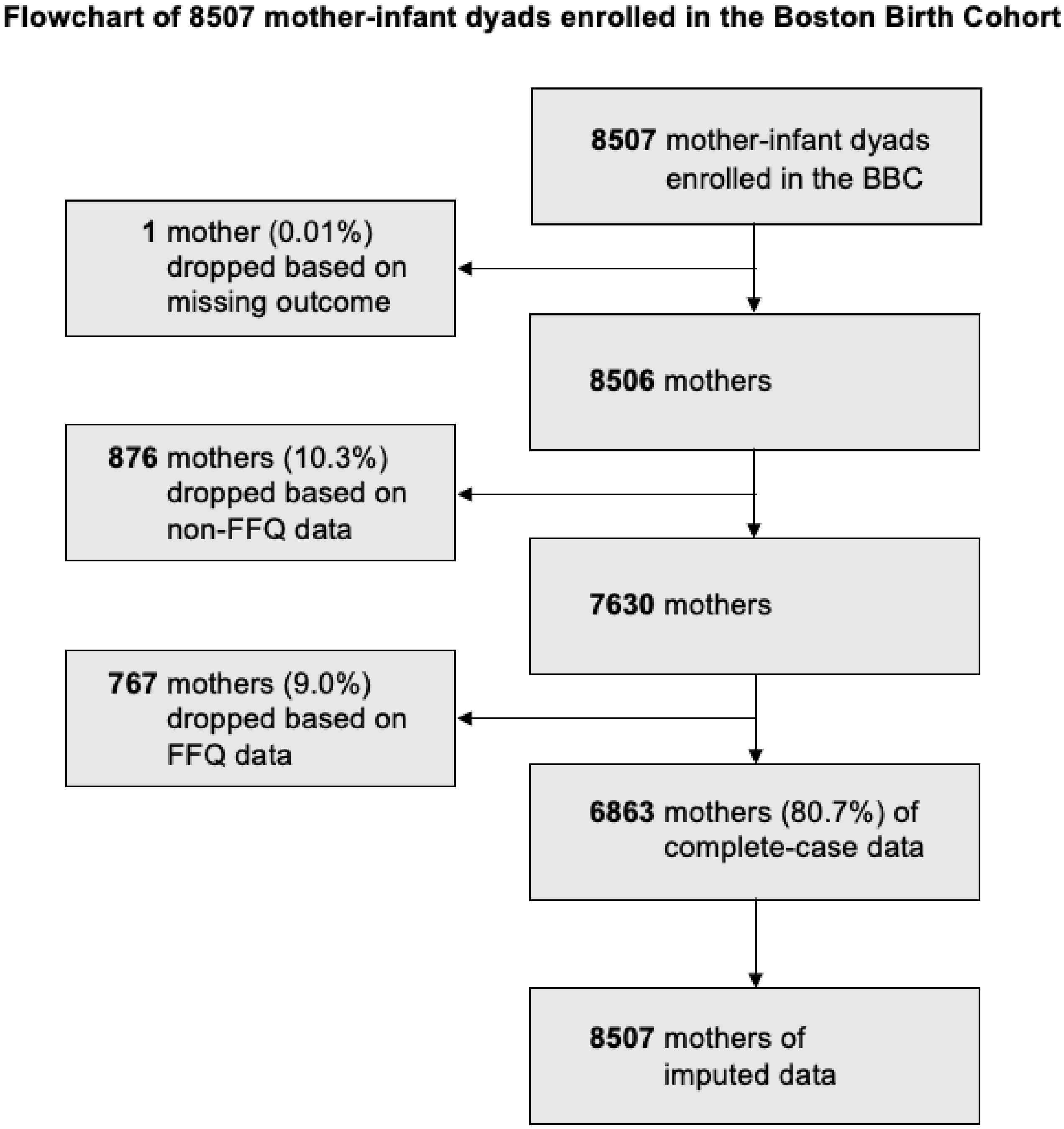

Figure S2. Flowchart of the full (raw), complete-case and imputed (final) analytical samples. Shown are the boxes containing the number of mothers at each step of the data-cleaning process as well as the percentage remaining compared to the full sample, where applicable. The initial full sample contained 8,507 mothers which was reduced to 8,506 after dropping one observation with a missing outcome. 7,630 remained following the next round of dropping of those with any missing response pertaining to the non-FFQ data. After dropping those with any missing response related to FFQ data, a complete-case sample of 6,863 mothers remained. The final analytical sample consisted of this complete-case sample plus 1,644 mothers whose missing values were imputed as described in the Materials and Methods section.

Table S1. Baseline characteristics of mothers in the Boston Birth Cohort, overall sample and stratified by MSDS quintiles (original data).

	N (%)					
**Baseline Characteristics**	**Total**	**Quintile 1**	**Quintile 2**	**Quintile 3**	**Quintile 4**	**Quintile 5**
**No.**	8,507	2,173 (25.5)	1.481 (17.4)	1.832 (21.5)	1,536 (18.1)	1,485 (17.5)
**Maternal age (years)** #						
<21	1,270 (14.9)	440 (20.3)	227 (15.3)	293 (16.0)	168 (10.9)	142 (9.6)
21-30	3,940 (46.3)	1,042 (48.0)	703 (47.5)	845 (46.1)	710 (46.2)	640 (43.1)
>30	3,297 (38.8)	691 (31.8)	551 (37.2)	694 (37.9)	658 (42.8)	703 (47.3)
**Race or ethnicity** #						
African American *	4,030 (47.4)	1,021 (47.0)	719 (48.6)	848 (46.3)	750 (48.8)	692 (46.6)
Non-Hispanic White	1,005 (11.8)	395 (18.2)	176 (11.9)	194 (10.6)	135 (8.8)	105 (7.1)
Hispanic	2,423 (28.5)	535 (24.6)	423 (28.6)	575 (31.4)	460 (30.0)	430 (29.0)
Other †	1,049 (12.3)	222 (10.2)	163 (11.0)	215 (11.7)	191 (12.4)	258 (17.4)
**Education Level** #						
Less than HS	2,629 (30.9)	759 (34.9)	445 (30.1)	599 (32.7)	443 (28.8)	383 (25.8)
HS or equivalent	2,793 (32.8)	731 (33.6)	499 (33.7)	577 (31.5)	498 (32.4)	488 (32.9)
Greater than HS	2,925 (34.4)	653 (30.1)	511 (34.5)	616 (33.6)	558 (36.3)	587 (39.5)
Missing	160 (1.9)	30 (1.4)	26 (1.8)	40 (2.2)	37 (2.4)	27 (1.8)
**Child's sex**						
Female	4,272 (50.2)	1,114 (51.3)	750 (50.6)	898 (49.0)	761 (49.5)	749 (50.4)
Male	4,235 (49.8)	1,059 (48.7)	731 (49.4)	934 (51.0)	775 (50.5)	736 (49.6)
**Ever smoked during pregnancy** #						
No	6,804 (80.0)	1,500 (69.0)	1,206 (81.4)	1,487 (81.2)	1,303 (84.8)	1,308 (88.1)
Yes	1,635 (19.2)	663 (30.5)	265 (17.9)	326 (17.8)	211 (13.7)	170 (11.5)
Missing	68 (0.8)	10 (0.5)	10 (0.7)	19 (1.0)	22 (1.4)	7 (0.5)
**Parity** ǁ						
0	3,661 (43.0)	986 (45.4)	621 (41.9)	806 (44.0)	624 (40.6)	624 (42.0)
>0	4,846 (57.0)	1,187 (54.6)	860 (58.1)	1,026 (56.0)	912 (59.4)	861 (58.0)
**Pre-pregnancy BMI (kg/m²)** #						
<18.5	370 (4.4)	111 (5.1)	74 (5.0)	68 (3.7)	69 (4.5)	48 (3.2)
18.5-24.9	3,797 (44.6)	939 (43.2)	642 (43.4)	850 (46.4)	681 (44.3)	685 (46.1)
25-29.9	2,155 (25.3)	530 (24.4)	387 (26.1)	459 (25.1)	417 (27.2)	362 (24.4)
≥30	1,606 (18.9)	474 (21.8)	284 (19.2)	339 (18.5)	265 (17.3)	244 (16.4)
Missing	579 (6.8)	119 (5.5)	94 (6.4)	116 (6.3)	104 (6.8)	146 (9.8)
**Diabetes** ǁ						
None	7,615 (89.5)	1,902 (87.5)	1,337 (90.3)	1,648 (90.0)	1,396 (90.9)	1,332 (89.7)
GDM	576 (6.8)	169 (7.8)	93 (6.3)	112 (6.1)	99 (6.5)	103 (6.9)
DM	307 (3.6)	101 (4.7)	50 (3.4)	70 (3.8)	39 (2.5)	47 (3.2)
Missing	9 (0.1)	1 (0.1)	1 (0.1)	2 (0.1)	2 (0.1)	3 (0.2)
**Chronic hypertension prior to pregnancy**						
No	8,006 (94.1)	2,041 (93.9)	1,378 (93.1)	1,740 (95.0)	1,444 (94.0)	1,403 (94.5)
Yes	442 (5.2)	118 (5.4)	95 (6.4)	75 (4.1)	83 (5.4)	71 (4.8)
Missing	59 (0.7)	14 (0.6)	8 (0.5)	17 (0.9)	9 (0.6)	11 (0.7)
**Preeclampsia** ǁ						
None	7,633 (89.7)	1,930 (88.8)	1,324 (89.4)	1,663 (90.8)	1,379 (89.8)	1,337 (90.0)
Mild	317 (3.7)	74 (3.4)	59 (4.0)	71 (3.9)	68 (4.4)	45 (3.0)
Severe	515 (6.1)	160 (7.4)	88 (5.9)	85 (4.6)	84 (5.5)	98 (6.6)
Missing	42 (0.5)	9 (0.4)	10 (0.7)	13 (0.7)	5 (0.3)	5 (0.3)
**Eclampsia**						
No	8,458 (99.4)	2,159 (99.4)	1,471 (99.3)	1,823 (99.5)	1,530 (99.6)	1,475 (99.3)
Yes	18 (0.2)	5 (0.2)	3 (0.2)	4 (0.2)	1 (0.1)	5 (0.3)
Missing	31 (0.4)	9 (0.4)	7 (0.5)	5 (0.3)	5 (0.3)	5 (0.3)
**HELLP syndrome**						

No	8,327 (97.9)	2,131 (98.1)	1,460 (98.6)	1,790 (97.7)	1,494 (97.3)	1,452 (97.8)
Yes	65 (0.8)	18 (0.8)	4 (0.3)	13 (0.7)	15 (1.0)	15 (1.0
Missing	115 (1.4)	24 (1.1)	17 (1.2)	29 (1.6)	27 (1.8)	18 (1.2)

Abbreviations: HS, high school; BMI, body mass index; GDM, gestational diabetes mellitus; DM, diabetes mellitus. Percentages may not add to 100% due to rounding.

* Included African Americans and Haitians.

† Included Asian, Cape Verdian, Pacific Islander, mixed-race, and others.

p-values (x^2 test): | | (≤ 0.05); ¶ (< 0.01); # (<0.001).

Table S2. Baseline characteristics of mothers in the Boston Birth Cohort, overall sample and stratified by MSDS quintiles (complete-case data).

	N (%)					
**Baseline Characteristics**	**Total**	**Quintile 1**	**Quintile 2**	**Quintile 3**	**Quintile 4**	**Quintile 5**
**No.**	6,863	1,426 (20.8)	1,638 (23.9)	1,476 (21.5)	1,180 (17.2)	1,143 (16.7)
**Maternal age (years)** #						
< 21	1,018 (14.8)	305 (21.4)	258 (15.8)	225 (15.2)	123 (10.4)	107 (9.4)
21–30	3,184 (46.4)	679 (47.6)	771 (47.1)	686 (46.5)	556 (47.1)	492 (43.0)
> 30	2,661 (38.8)	442 (31.0)	609 (37.2)	565 (38.3)	501 (42.5)	544 (47.6)
**Race or ethnicity** #						
African American *	3,221 (46.9)	680 (47.7)	779 (47.6)	671 (45.5)	560 (47.5)	531 (46.5)
Non-Hispanic White	828 (12.1)	268 (18.8)	204 (12.5)	155 (10.5)	110 (9.3)	91 (8.0)
Hispanic	1,950 (28.4)	332 (23.3)	474 (28.9)	464 (31.4)	361 (30.6)	319 (27.9)
Other †	864 (12.6)	146 (10.2)	181 (11.1)	186 (12.6)	149 (12.6)	202 (17.7)
**Education Level** #						
Less than HS	2,088 (30.4)	519 (36.4)	494 (30.2)	464 (31.4)	331 (28.1)	280 (24.5)
HS or equivalent	2,273 (33.1)	478 (33.5)	567 (34.6)	470 (31.8)	383 (32.5)	375 (32.8)
Greater than HS	2,502 (36.5)	429 (30.1)	577 (35.2)	542 (36.7)	466 (39.5)	488 (42.7)
**Child's sex**						
Female	3,437 (50.1)	715 (50.1)	840 (51.3)	719 (48.7)	586 (49.7)	577 (50.5)
Male	3,426 (49.9)	711 (49.9)	798 (48.7)	757 (51.3)	594 (50.3)	566 (49.5)
**Ever smoked during pregnancy** #						
No	5,461 (79.6)	950 (66.6)	1,301 (79.4)	1,211 (82.1)	1,004 (85.1)	995 (87.1)
Yes	1,402 (20.4)	476 (33.4)	337 (20.6)	265 (18.0)	176 (14.9)	148 (13.0)
**Parity**						
0	2,995 (43.6)	655 (45.9)	703 (42.9)	648 (43.9)	498 (42.2)	491 (43.0)
> 0	3,868 (56.4)	771 (54.1)	935 (57.1)	828 (56.1)	682 (57.8)	652 (57.0)
**Pre-pregnancy BMI (kg/m²)** ¶						
< 18.5	320 (4.7)	84 (5.9)	81 (5.0)	63 (4.3)	52 (4.4)	40 (3.5)
18.5–24.9	3,293 (48.0)	657 (46.1)	763 (46.6)	720 (48.8)	561 (47.5)	592 (51.8)
25–29.9	1,857 (27.1)	356 (25.0)	456 (27.8)	404 (27.4)	341 (28.9)	300 (26.3)
≥ 30	1,393 (20.3)	329 (23.1)	338 (20.6)	289 (19.6)	226 (19.2)	211 (18.5)
**Diabetes**						
None	6,127 (89.3)	1,252 (87.8)	1,465 (89.4)	1,323 (89.6)	1,062 (90.0)	1,025 (89.7)
GDM	474 (6.9)	106 (7.4)	112 (6.8)	92 (6.2)	87 (7.4)	77 (6.7)
DM	262 (3.8)	68 (4.8)	61 (3.7)	61 (4.1)	31 (2.6)	41 (3.6)
**Chronic hypertension prior to pregnancy**						
No	6,496 (94.7)	1,352 (94.8)	1,536 (93.8)	1,411 (95.6)	1,116 (94.6)	1,081 (94.6)
Yes	367 (5.4)	74 (5.2)	102 (6.2)	65 (4.4)	64 (5.4)	62 (5.4)
**Preeclampsia** ¶						
None	6,194 (90.3)	1,278 (89.6)	1,471 (89.8)	1,349 (91.4)	1,070 (90.7)	1,026 (89.8)
Mild	247 (3.6)	45 (3.2)	60 (3.7)	62 (4.2)	48 (4.1)	32 (2.8)
Severe	422 (6.2)	103 (7.2)	107 (6.5)	65 (4.4)	62 (5.3)	85 (7.4)
**Eclampsia**						
No	6,847 (99.8)	1,423 (99.8)	1,634 (99.8)	1,473 (99.8)	1,179 (99.9)	1,138 (99.6)
Yes	16 (0.2)	3 (0.2)	4 (0.2)	3 (0.2)	1 (0.1)	5 (0.4)
**HELLP syndrome**						
No	6,811 (99.2)	1,416 (99.3)	1,630 (99.5)	1,466 (99.3)	1,171 (99.2)	1,128 (98.7)
Yes	52 (0.8)	10 (0.7)	8 (0.5)	10 (0.7)	9 (0.8)	15 (1.3)

Abbreviations: HS, high school; BMI, body mass index; GDM, gestational diabetes mellitus; DM, diabetes mellitus. Percentages may not add to 100% due to rounding.

* Included African Americans and Haitians.

† Included Asian, Cape Verdian, Pacific Islander, mixed-race, and others.

p-values (x^2 test): ǀǀ (≤ 0.05); ¶ (< 0.01); # (<0.001).

Table S3. Adjusted stratified analyses on the association between MSDS quintiles and birth outcomes in the Boston Birth Cohort, relative to quintile 5 (overall sample).

Outcome	Relative risk (95% CI)			
	**Quintile 1**	**Quintile 2**	**Quintile 3**	**Quintile 4**
< 37 weeks				
**Maternal age (years)**				
< 21	1.52 (1.07 – 2.16) | |	1.42 (0.98 – 2.07)	1.01 (0.69 – 1.49)	0.95 (0.61 – 1.48)
21–30	1.21 (1.01 – 1.44) | |	1.14 (0.95 – 1.39)	1.07 (0.88 – 1.29)	1.14 (0.94 – 1.38)
> 30	1.09 (0.94 – 1.27)	0.89 (0.75 – 1.06)	0.89 (0.76 – 1.05)	1.01 (0.86 – 1.18)
**Education level**				
Less than HS	1.16 (0.95 – 1.42)	1.08 (0.86 – 1.35)	0.93 (0.74 – 1.16)	0.97 (0.77 – 1.22)
HS or equivalent	1.20 (1.00 – 1.43) | |	0.97 (0.80 – 1.19)	0.97 (0.80 – 1.17)	1.12 (0.93 – 1.35)
Greater than HS	1.16 (0.97 – 1.39)	1.09 (0.90 – 1.33)	0.96 (0.79 – 1.16)	1.02 (0.84 – 1.23)
**Ever smoked during Pregnancy**				
No	1.17 (1.04 – 1.32) | |	1.02 (0.90 – 1.17)	0.99 (0.87 – 1.13)	1.03 (0.90 – 1.17)
Yes	1.17 (0.93 – 1.49)	1.09 (0.83 – 1.42)	0.85 (0.64 – 1.11)	1.12 (0.85 – 1.48)
**Parity**				
0	1.23 (1.04 – 1.45) | |	1.16 (0.97 – 1.40)	1.03 (0.86 – 1.23)	1.09 (0.90 – 1.31)
> 0	1.15 (1.00 – 1.32) | |	0.98 (0.84 – 1.14)	0.91 (0.79 – 1.07)	1.02 (0.88 – 1.18)
**Pre-pregnancy BMI (kg/m^2)**				
< 25	1.16 (0.99 – 1.36)	1.19 (1.01 – 1.41) | |	0.89 (0.74 – 1.06)	1.07 (0.90 – 1.27)
≥ 25	1.19 (1.04 – 1.38)	0.93 (0.79 – 1.09)	1.04 (0.89 – 1.21)	1.03 (0.88 – 1.21)
**Diabetes**				
No	1.18 (1.05 – 1.33) ¶	1.06 (0.93 – 1.20)	0.96 (0.85 – 1.09)	1.01 (0.89 – 1.15)
Yes	1.16 (0.90 – 1.50)	0.98 (0.73 – 1.32)	0.94 (0.71 – 1.26)	1.26 (0.95 – 1.66)
**Blood pressure complication during pregnancy**				
No	1.27 (1.11 – 1.45) #	1.14 (0.99 – 1.32)	0.99 (0.86 – 1.14)	1.12 (0.97 – 1.29)
Yes	0.95 (0.81 – 1.11)	0.82 (0.68 – 0.98) | |	0.89 (0.75 – 1.06)	0.85 (0.71 – 1.02)
Spontaneous preterm birth				
**Maternal age (years)**				
< 21	1.54 (1.01 – 2.35) | |	1.49 (0.95 – 2.34)	1.11 (0.70 – 1.76)	1.05 (0.63 – 1.75)
21–30	1.41 (1.11 – 1.78) ¶	1.24 (0.96 – 1.61)	1.13 (0.87 – 1.45)	1.28 (0.99 – 1.66)
> 30	1.12 (0.90 – 1.39)	0.90 (0.71 – 1.16)	1.01 (0.80 – 1.26)	1.05 (0.84 – 1.31)
**Education level**				
Less than HS	1.14 (0.87 – 1.50)	1.08 (0.80 – 1.50)	0.99 (0.75 – 1.33)	1.07 (0.79 – 1.45)
HS or equivalent	1.40 (1.10 – 1.78)	1.12 (0.85 – 1.47)	1.08 (0.83 – 1.41)	1.23 (0.94 – 1.60)
Greater than HS	1.27 (0.99 – 1.64)	1.12 (0.86 – 1.48)	1.05 (0.81 – 1.37)	1.09 (0.83 – 1.42)
**Ever smoked during Pregnancy**				
No	1.27 (1.07 – 1.51) ¶	1.10 (0.91 – 1.32)	1.10 (0.92 – 1.31)	1.10 (0.92 – 1.32)
Yes	1.23 (0.91 – 1.67)	1.10 (0.78 – 1.56)	0.89 (0.62 – 1.26)	1.24 (0.87 – 1.75)
**Parity**				
0	1.40 (1.11 – 1.76) ¶	1.32 (1.03 – 1.70) | |	1.16 (0.91 – 1.48)	1.23 (0.96 – 1.58)
> 0	1.20 (1.00 – 1.46)	0.98 (0.79 – 1.22)	0.98 (0.80 – 1.21)	1.07 (0.87 – 1.32)
**Pre-pregnancy BMI (kg/m2)**				
< 25	1.26 (1.02 – 1.55) | |	1.29 (1.03 – 1.60) | |	0.96 (0.76 – 1.20)	1.17 (0.94 – 1.47)
≥ 25	1.30 (1.06 – 1.60) | |	0.93 (0.73 – 1.18)	1.16 (0.93 – 1.44)	1.10 (0.88 – 1.38)
**Diabetes**				
No	1.26 (1.08 – 1.47) ¶	1.09 (0.92 – 1.30)	1.03 (0.88 – 1.22)	1.09 (0.92 – 1.29)
Yes	1.47 (0.95 – 2.28)	1.27 (0.77 – 2.12)	1.20 (0.73 – 1.97)	1.56 (0.97 – 2.53)
**Blood pressure complication during pregnancy**				
No	1.32 (1.13 – 1.53) #	1.16 (0.98 – 1.37)	1.03 (0.88 – 1.22)	1.19 (1.01 – 1.40)

Yes	1.00 (0.60 – 1.68)	0.73 (0.39 – 1.36)	1.30 (0.77 – 2.21)	0.63 (0.33 – 1.20)
34–36 weeks				
**Maternal age (years)**				
< 21	1.70 (1.07 – 2.71) ‖	1.66 (1.01 – 2.70) ‖	0.97 (0.58 – 1.63)	1.16 (0.67 – 2.02)
21–30	1.16 (0.93 – 1.45)	1.09 (0.86 – 1.39)	1.02 (0.81 – 1.30)	1.09 (0.85 – 1.38)
> 30	1.15 (0.94 – 1.41)	0.89 (0.70 – 1.12)	0.88 (0.70 – 1.10)	1.03 (0.83 – 1.28)
**Education level**				
Less than HS	1.08 (0.84 – 1.39)	1.04 (0.79 – 1.37)	0.82 (0.62 – 1.09)	0.99 (0.74 – 1.31)
HS or equivalent	1.35 (1.06 – 1.71) ‖	1.00 (0.76 – 1.31)	0.96 (0.74 – 1.25)	1.22 (0.95 – 1.58)
Greater than HS	1.16 (0.91 – 1.48)	1.11 (0.86 – 1.44)	1.00 (0.77 – 1.28)	0.94 (0.72 – 1.23)
**Ever smoked during Pregnancy**				
No	1.21 (1.03 – 1.41) ‖	1.02 (0.85 – 1.21)	0.97 (0.82 – 1.15)	1.02 (0.86 – 1.21)
Yes	1.18 (0.86 – 1.62)	1.14 (0.80 – 1.62)	0.79 (0.55 – 1.15)	1.23 (0.86 – 1.77)
**Parity**				
0	1.27 (1.02 – 1.58) ‖	1.13 (0.89 – 1.45)	1.03 (0.81 – 1.31)	1.16 (0.92 – 1.48)
> 0	1.16 (0.97 – 1.40)	1.00 (0.82 – 1.22)	0.86 (0.71 – 1.06)	1.00 (0.81 – 1.22)
**Pre-pregnancy BMI (kg/m2)**				
< 25	1.15 (0.94 – 1.41)	1.23 (1.00 – 1.53)	0.85 (0.68 – 1.06)	1.06 (0.85 – 1.32)
≥ 25	1.26 (1.04 – 1.53)	0.88 (0.70 – 1.11)	1.03 (0.83 – 1.27)	1.07 (0.87 – 1.33)
**Diabetes**				
No	1.19 (1.02 – 1.39)	1.05 (0.89 – 1.24)	0.93 (0.79 – 1.10)	1.01 (0.86 – 1.20)
Yes	1.29 (0.90 – 1.87)	1.03 (0.68 – 1.58)	0.91 (0.59 – 1.39)	1.40 (0.94 – 2.08)
**Blood pressure complication during pregnancy**				
No	1.32 (1.12 – 1.56) ¶	1.17 (0.97 – 1.41)	0.99 (0.83 – 1.19)	1.17 (0.97 – 1.40)
Yes	0.88 (0.69 – 1.13)	0.72 (0.54 – 0.95) ‖	0.76 (0.58 – 1.01)	0.74 (0.56 – 0.99) ‖)
Low birth weight				
**Maternal age (years)**				
< 21	1.22 (0.94 – 1.57)	1.21 (0.91 – 1.60)	1.32 (1.01 – 1.72) ‖	0.88 (0.60 – 1.30)
21–30	1.13 (0.97 – 1.32)	1.24 (1.05 – 1.46) ‖	1.02 (0.86 – 1.20)	1.03 (0.87 – 1.23)
> 30	1.08 (0.94 – 1.24)	1.09 (0.94 – 1.26)	1.05 (0.91 – 1.22)	1.07 (0.93 – 1.22)
**Education level**				
Less than HS	1.09 (0.91 – 1.30)	1.15 (0.95 – 1.30)	1.05 (0.87 – 1.27)	0.89 (0.72 – 1.11)
HS or equivalent	1.12 (0.96 – 1.31)	1.19 (1.01 – 1.41) ‖	1.17 (0.99 – 1.38)	1.03 (0.87 – 1.22)
Greater than HS	1.13 (0.96 – 1.32)	1.15 (0.97 – 1.36)	0.98 (0.82 – 1.16)	1.16 (0.99 – 1.38)
**Ever smoked during Pregnancy**				
No	1.10 (0.99 – 1.23)	1.16 (1.04 – 1.30) ‖	1.07 (0.96 – 1.19)	1.02 (0.91 – 1.15)
Yes	1.13 (0.92 – 1.37)	1.15 (0.93 – 1.43)	1.04 (0.83 – 1.30)	1.06 (0.84 – 1.34)
**Parity**				
0	1.14 (0.99 – 1.30)	1.25 (1.08 – 1.45) ¶	1.02 (0.88 – 1.18)	0.95 (0.81 – 1.12)
> 0	1.09 (0.96 – 1.25)	1.09 (0.95 – 1.26)	1.12 (0.98 – 1.28)	1.10 (0.96 – 1.26)
**Pre-pregnancy BMI (kg/m2)**				
< 25	1.23 (1.08 – 1.41) ¶	1.23 (1.06 – 1.42) ¶	1.13 (0.98 – 1.30)	1.06 (0.91 – 1.23)
≥ 25	1.00 (0.87 – 1.14)	1.12 (0.97 – 1.29)	1.00 (0.87 – 1.15)	1.01 (0.88 – 1.16)
**Diabetes**				
No	1.14 (1.04 – 1.26) ¶	1.17 (1.05 – 1.30) ¶	1.09 (0.98 – 1.21)	1.05 (0.94 – 1.17)
Yes	0.89 (0.66 – 1.21)	1.19 (0.88 – 1.61)	0.95 (0.69 – 1.31)	0.95 (0.68 – 1.33)
**Blood pressure complication during pregnancy**				
No	1.09 (0.98 – 1.23)	1.18 (1.04 – 1.33) ‖	1.07 (0.95 – 1.21)	0.99 (0.87 – 1.13)
Yes	1.13 (0.98 – 1.31)	1.08 (0.92 – 1.27)	1.05 (0.89 – 1.22)	1.11 (0.95 – 1.31)

Abbreviations: HS, high school; BMI, body mass index.

Covariates adjusted for are: maternal age, race, educational level, smoking status, parity, pre-pregnancy BMI, diabetes status, and blood pressure complications during pregnancy. Continuous birth weight outcome was derived additionally adjusting for the gestational age, and low birth weight outcome was derived additionally adjusting for the overall preterm birth. Log-binomial regression was used to derive the relative risk.

P-values (x^2 test): | | (≤ 0.05); ¶ (< 0.01); # (< 0.001).
